# Progressive resolution optimizer (PRO) predominates over photon optimizer (PO) in sparing of spinal cord for spine SABR VMAT plans

**DOI:** 10.1186/s12885-023-10925-z

**Published:** 2023-05-16

**Authors:** Sangjun Son, So-Yeon Park

**Affiliations:** 1 Department of Radiation Oncology, Seoul National University Hospital, Seoul, Republic of Korea; 2 Department of Radiation Oncology, Veterans Health Service Medical Center, Seoul, Republic of Korea; 3 Institute of Radiation Medicine, Seoul National University Medical Research Center, Seoul, Republic of Korea

**Keywords:** Progressive resolution optimizer, Photon optimizer, Spine, Stereotactic ablative radiation therapy, Volumetric modulated arc therapy

## Abstract

**Background:**

we assessed the performance of the optimization algorithms by comparing volumetric modulated arc therapy generated by a progressive resolution optimized (VMAT_PRO_) and photon optimizer (VMAT_PO_) in terms of plan quality, MU reduction, sparing of the spinal cord (or cauda equina), and plan complexity.

**Methods:**

Fifty-seven patients who received spine stereotactic ablative radiotherapy (SABR) with tumors located in the cervical, thoracic, and lumbar spine were retrospectively selected. For each patient, VMAT_PRO_ and VMAT_PO_ with two full arcs were generated with using the PRO and PO algorithms. For dosimetric evaluation, the dose-volumetric (DV) parameters of the planning target volume (PTV), organs at risk (OARs), the corresponding planning organs at risk (PRV), and 1.5-cm ring structure surrounding the PTV (Ring_1.5 cm_) were calculated for all VMAT plans. The total number of monitor units (MUs) and the modulation complexity score for the VMAT (MCS_v_) were compared. To investigate the correlations of OAR sparing to plan complexity, Pearson’s and Spearman’s correlation tests were conducted between the two algorithms (PO – PRO, denoted as Δ) in the DV parameters for normal tissues, total MUs, and MCS_v_.

**Results:**

For the PTVs, Target conformity and dose homogeneity in the PTVs of VMAT_PRO_ were better than those of VMAT_PO_ with statistical significance. For the spinal cords (or cauda equine) and the corresponding PRVs, all of the DV parameters for VMAT_PRO_ were markedly lower than those for VMAT_PO_, with statistical significance (all *p* < 0.0001). Among them, the difference in the maximum dose to the spinal cord between VMAT_PRO_ and VMAT_PO_ was remarkable (9.04 Gy vs. 11.08 Gy with *p* < 0.0001). For Ring_1.5 cm_, no significant difference in V_115%_ for VMAT_PRO_ and VMAT_PO_ was observed.

**Conclusions:**

The use of VMAT_PRO_ resulted in improved coverage and uniformity of dose to the PTV, as well as OARs sparing, compared with that of VMAT_PO_ for cervical, thoracic, and lumbar spine SABR. Better dosimetric plan quality generated by the PRO algorithm was observed to result in higher total MUs and plan complexity. Therefore, careful evaluation of its deliverability should be performed with caution during the routine use of the PRO algorithm.

**Supplementary Information:**

The online version contains supplementary material available at 10.1186/s12885-023-10925-z.

## Background

Bone metastases occur in approximately one-third of all patients with advanced malignant cancers, of which 70% originate within the spine [1–4]. Radiotherapy has been the standard treatment for decades for patients with spinal metastasis not requiring or amenable to surgery [5]. With the rapid development of technology and equipment, stereotactic ablative radiotherapy (SABR) can deliver a high dose in a few fractions (one–five fractions) with a steep dose fall-off, providing a high biologically equivalent dose to the target volume and sparing nearby normal organs adjacent to the target volume. Several studies have shown that SABR for spinal metastasis is more effective for local tumor control and pain relief than traditional radiotherapy [6–10].

Conversely, other studies reported that radiation myelopathy, the most morbid complication associated with spine SABR, has been observed [11, 12]. The risk of radiation myelopathy is low, but it can have a huge negative impact on the quality of life and prognosis. Symptoms can include difficulty walking, numbness, limb weakness, loss of bladder and bowel control, and death [11, 12]. Therefore, to prevent radiation myelopathy, it is important to reduce the dose to the spinal cord and cauda equina as much as possible. Thus, an extremely rapid dose fall-off between the spine and spinal cord should be achieved because the spinal cord is surrounded by irregular vertebral bodies and the target volumes for spinal metastasis are irregularly shaped.

In this regard, volumetric modulated arc therapy (VMAT) with varying gantry speeds, dose rates, and multi-leaf collimator (MLC) speeds is a suitable treatment option for spine SABR. These modulations of VMAT can generate steep dose gradients between target volumes and organs at risk (OARs) and provide highly conformal target coverage within a shorter treatment time, compared with the intensity modulated radiotherapy (IMRT) technique [13–15]. For the generation of VMAT plans, an inverse optimization process that determines the combination of field shapes and segment weights has been used based on dose-volume histogram (DVH) information. However, this method leaves little room for user intervention during the optimization process. Therefore, the dosimetric quality of the VMAT plans is highly dependent on the performance of optimization algorithms in the treatment planning system (TPS).

Recently, Varian Eclipse TPS (Varian Medical Systems, Palo Alto, CA, version 13.5) introduced a new optimization algorithm called the photon optimizer (PO). The PO algorithm can be used for both IMRT and VMAT plans, whereas the dose-volume optimizer (DVO) and progressive resolution optimizer (PRO) from the previous version of Eclipse were used for IMRT and VMAT plan generation, respectively. The main difference of the PRO algorithm from the PO algorithm is that the PO algorithm uses a new structure model, where the structure, DVH calculations, and dose sampling are defined spatially by using a single matrix over the image instead of a point cloud model that is used in PRO algorithm [16–18]. User-specified fixed values (1.25 mm, 2.5 mm, or 5 mm) are used for the voxel resolution of the matrix [16–18]. For fast dose estimation during optimization, both the PRO and PO algorithms utilize a multiresolution dose calculation algorithm that go through four multi-resolution levels, and include the intermediate dose calculation option to acquire better dosimetric plan quality [16, 18].

Several studies have analyzed the dosimetric impact, treatment efficiency, and plan complexity between various plans generated by the PRO and PO algorithms for various sites. Liu et al. demonstrated that the PO algorithm showed comparable plan quality and less plan complexity with fewer total monitor units (MUs) for VMAT planning of both lung SABR and brain stereotatic radiosurgery (SRS) [17]. Other institutions have also shown the superiority of the PO algorithm over the PRO algorithm in terms of treatment efficiency (MU reduction) without compromising the VMAT plan quality for lung SABR [18, 19]. However, some studies have reported contradictory results for the PO algorithm. Binny et al. investigated the plan quality of intensity-modulated arc therapy for the prostate, head and neck, and brain treatment sites [16]. They observed that plans optimized using the PO algorithm had higher MLC complexity and higher total MUs, while improving OAR sparing with a similar degree of dose conformity to the target volume, compared with those optimized using the PRO algorithm [16]. Kim et al. yielded conflicting results for IMRT and VMAT planning techniques [20]. Although prostate IMRT and VMAT plans generated using the PO algorithm showed an improvement in plan quality for the target volume over the DVO and PRO algorithms, total MU reductions for the PO algorithm were observed only in the IMRT plans, whereas more total MUs for the PO algorithm were used in the VMAT plans [20]. Therefore, the superiority of the PO algorithm is not obvious and varies based on the radiotherapy regimen used and the treatment site. To the best of our knowledge, no planning study of VMAT for spine SABR generated using the PRO and PO algorithms has been performed. In this study, we assessed the performance of the optimization algorithms by comparing PRO-generated VMAT plans (VMAT_PRO_) with PO-generated VMAT plans (VMAT_PO_) in terms of plan quality, MU reduction, sparing of the spinal cord (or cauda equina), and plan complexity. We included 57 patients who received spine SABR with tumors located in the cervical, thoracic, and lumbar spine.

## Methods

### Patient selection, simulation, and contouring

From January 2016 to September 2020, 57 patients with spinal metastasis who had a single target volume were retrospectively chosen at our institution. Twenty-eight patients with cervical or thoracic spinal metastases and 29 patients with lumbar spinal metastasis were selected. All patients were previously treated with SABR using the VMAT technique. Approval for this study was obtained from the Institutional Review Board (IRB No. 2020-11-008). All patients underwent computed tomography (CT) scans using various immobilization techniques at the treatment sites using the Brilliance CT Big Bore™ (Philips, Amsterdam, Netherlands). CT images were acquired with 512 × 512 pixels at a 1-mm slice thickness.

The target volume of this study was the planning target volume (PTV). The clinical target volume (CTV) and OARs were defined by a single oncologist based on T1-weighted and T2 MR images. The OAR was selectively determined as the spinal cord or cauda equina, according to the tumor location. Normal organs, except the spinal cord and cauda equina, were not analyzed as OARs in this study. PTV and planning organ-at-risk volume (PRV) were generated by adding an isotropic margin of 1 mm from the CTV and OAR, respectively. For dosimetric evaluation and plan optimization, a 1.5-cm ring structure surrounding the PTV (Ring_1.5 cm_) was created. The PRV overlap inside the PTV was excluded from the PTV to spare more normal tissues, including the spinal cord and cauda equina.

### Treatment planning

Every VMAT plan was generated using 10 MV flattening filter-free photon beams from TrueBeam STx with a high-definition 120™ MLC (Varian Medical Systems, Palo Alto, CA, USA). Each VMAT plan consisted of two full arcs with collimator angles of 350° and 273°. All VMAT_PO_ were optimized with the PO algorithm of the Eclipse TPS (version 13.7, Varian Medical Systems, Palo Alto, CA, USA) using a fixed 2.5-mm voxel resolution. Additionally, the jaw-tracking option was employed to minimize the leakage dose to the normal tissues. The prescription dose of the PTV was 18 Gy in a single fraction of the spine SABR. During optimization, planning constraints of the Radiation Therapy Oncology Group (RTOG) 0631 study were followed to spare normal organs and avoid complications. Table 1 lists the planning constraints of the target volume, and OARs for the spine SABR. Conservatively, these constraints are applied to the corresponding PRVs. Automatic normal tissue optimization (NTO) with a priority of 300 was used. To improve the dosimetric plan quality, all VMAT_PO_ were reoptimized using the current dose distribution as a reference. Dose distributions were calculated using the Acuros XB advanced dose calculation algorithm (version 13.7, Varian Medical Systems, Palo Alto, CA, USA) with a calculation grid of 2 mm. Each plan was normalized such that at least 80% of the PTV received a prescribed dose.

**Table 1:** Planning constraints of planning target volume, normal organs and planning organ at risk volumes for spine stereotactic ablative radiotherapy plans

Structure	Planning constraints	Priority
PTV	D0% < 18.5 Gy	200
	D100% > 18 Gy	150
Spinal cord (or spinal cord PRV)	D_1.2 cc_ < 7 Gy	120
	D_0.35 cc_ < 10 Gy	120
	D_0.035 cc_ < 14 Gy	150
Cauda equine (or cauda equine PRV)	D_5 cc_ < 14 Gy	120
	D_0.035 cc_ < 16 Gy	150

Note: PTV = planning target volume; PRV = planning organ at risk volume;

For comparison, all VMAT_PRO_ were optimized with the PRO algorithm of Eclipse TPS (version 13.7) using the identical beam geometry and planning protocols. To investigate the variation due to the optimization algorithms separately, the same planning constraints, objectives, automatic NTO and priorities for the target volume and normal tissues were used for both VMAT_PRO_ and VMAT_PO_.

### Evaluation of treatment plan

The dose-volumetric (DV) parameters calculated from each plan were analyzed to evaluate the dosimetric quality with respect to the target coverage and dose received by normal organs. For the PTV, the evaluated DV parameters were the maximum dose, minimum dose, mean dose, and the dose received at least 98% volume of the target volume (D_98%_), D_90%_, D_5%_, and D_2%_ were calculated. The conformity index suggested by Paddick et al. (CI_paddick_) and the homogeneity index (HI) were calculated as follows [21–23]:

1 $${CI}_{paddick}=\frac{{TV}_{prescription dose}}{TV}\times \frac{{TV}_{prescription dose}}{{V}_{prescription dose}}$$

2 $$\text{H}\text{I}= \frac{{D}_{2\%}-{D}_{98\%}}{mean dose}$$

where *TV*_*prescription dose*_ is the target volume covered by the prescription dose, *TV* is the target volume, and *V*_*prescription dose*_ is the volume of the prescription dose.

For the spinal cord and the corresponding PRVs, the evaluated DV parameters were the maximum dose, mean dose, D_1.2 cc_, D_0.35 cc_, and D_0.035 cc_. For the cauda equina and the corresponding PRVs, the maximum dose, mean dose, D_1.5 cc_, D_0.5 cc_, D_0.1 cc_, and D_0.035 cc_ were evaluated as DV parameters. For the Ring_1.5 cm_, the volumes receiving at least 105% of the ring structure (V_105%_), V_110%_, and V_115%_ were calculated from each type of plan.

To assess treatment efficiency and deliverability, the total number of MUs and the modulation complexity score for the VMAT (MCS_v_) were compared. The MCS_v_ proposed by Masi et al. can evaluate the complexity of the MLC movement and beam aperture shape of the VMAT plans [24]. The value of MCS_v_ decreased as the modulation complexity increased. This metric for each plan was calculated using in-house software (MATLAB R2021a, MathWorks, Natick, MA, USA).

Based on the Shapiro-Wilk test for the normality of the two corresponding datasets, a paired t-test or Wilcoxon signed rank test was used for pairwise comparisons of the DV parameters, total MUs, and MCS_v_ between the PO and PRO algorithms. To investigate the correlations of OAR sparing to the level of modulations, we utilized the differences between the two algorithms (PO – PRO, denoted as Δ) in the DV parameters for normal tissues, total MUs, and MCS_v_. With these data, correlation coefficients and corresponding *p*-values were obtained by conducting Pearson’s and Spearman’s correlation tests for parametric and non-parametric data, respectively, and *p*-values were considered statistically significant at *p* < 0.05. All analyses were performed using the PRISM statistical program (version 8.4.3, GraphPad Software Inc., San Diego, CA, USA).

## Results

### Dose-volumetric (DV) parameters

Table 2 summarizes the average DV parameters of both VMAT_PRO_ and VMAT_PO_ for the cervical and thoracic spine cases. For the PTVs, the differences in all the DV parameters analyzed in this study between VMAT_PRO_ and VMAT_PO_ were statistically significant (*p* < 0.05), except for D_98%_ and minimum dose (*p* = 0.167 and 0.141, respectively). The values of D_5%_, D_2%_, maximum dose, and mean dose were lower for VMAT_PRO_ than for VMAT_PO_ while the values of D_90%_ were slightly higher for VMAT_PRO_ than for VMAT_PO_. Target conformity and dose homogeneity in the PTVs of VMAT_PRO_ were better than those of VMAT_PO_ with statistical significance (0.90 vs. 0.82 with *p* < 0.0001 for CI_paddick_ and 0.32 vs. 0.35 with *p* < 0.001 for HI). The overall quality of the DV parameters of the PTVs was superior in VMAT_PRO_ than those in VMAT_PO_.

**Table 2:** Average dose-volumetric parameters of the planning target volume, spinal cord, planning organ at risk volume of spinal cord, and 1.5-cm ring structure surrounding PTV for cervical and thoracic spine stereotactic ablative radiotherapy plans

DV parameter	PRO	PO	*p*-value
PTV			
Volume (cm^3^)	51.46 ± 40.92		-
D_98%_ (Gy)	13.50 ± 1.40	13.68 ± 1.16	0.167
D_90%_ (Gy)	17.01 ± 0.56	16.79 ± 0.51	< 0.0001
D_5%_ (Gy)	19.30 ± 0.35	19.98 ± 0.60	< 0.0001
D_2%_ (Gy)	19.43 ± 0.37	20.15 ± 0.62	< 0.0001
Maximum dose (Gy)	20.25 ± 0.66	20.99 ± 0.83	< 0.0001
Minimum dose (Gy)	8.71 ± 1.44	8.40 ± 1.85	0.141
Mean dose (Gy)	18.29 ± 0.14	18.63 ± 0.28	< 0.0001
CI	0.90 ± 0.07	0.82 ± 0.10	< 0.0001
HI	0.32 ± 0.09	0.35 ± 0.08	< 0.001
Spinal cord			
Volume (cm^3^)	2.51 ± 0.72		-
D_1.2 cc_ (Gy)	4.52 ± 0.92	5.37 ± 1.44	< 0.0001
D_0.35 cc_ (Gy)	6.51 ± 0.79	8.18 ± 1.20	< 0.0001
D_0.035 cc_ (Gy)	7.86 ± 0.77	9.88 ± 1.13	< 0.0001
Maximum dose (Gy)	9.04 ± 0.79	11.08 ± 1.20	< 0.0001
Mean dose (Gy)	4.58 ± 0.60	5.52 ± 0.93	< 0.0001
Spinal cord PRV			
Volume (cm^3^)	5.07 ± 1.38		
D_1.2 cc_ (Gy)	7.26 ± 0.86	8.55 ± 1.25	< 0.0001
D_0.35 cc_ (Gy)	9.26 ± 0.70	10.78 ± 1.12	< 0.0001
D_0.035 cc_ (Gy)	10.88 ± 0.65	12.59 ± 1.09	< 0.0001
Maximum dose (Gy)	12.25 ± 0.86	13.83 ± 1.25	< 0.0001
Mean dose (Gy)	5.31 ± 0.64	6.11 ± 0.94	< 0.0001
Ring_1.5 cm_			
V_105%_ (cm^3^)	0.44 ± 0.61	2.66 ± 3.14	< 0.001
V_110%_ (cm^3^)	0.02 ± 0.06	0.63 ± 1.50	0.039
V_115%_ (cm^3^)	0.00 ± 0.00	0.13 ± 0.45	0.154

Note: DV = dose-volumetric; PRO = progressive resolution optimizer algorithm; PO = photon optimizer algorithm; PTV = planning target volume; D_n%_ = dose received by at least *n*% volume of the planning target volume; CI = conformity index; HI = homogeneity index; D_n cc_ = dose received by at least *n* cc volume of the planning target volume; PRV = planning organ at risk volume; V_n%_ = absolute volume of a structure irradiated by at least *n*% of the prescription dose; Ring_1.5 cm_ = 1.5-cm ring structure surrounding PTV

For the spinal cords and corresponding PRVs, all of the DV parameters for VMAT_PRO_ were markedly lower than those for VMAT_PO_, with statistical significance (all *p* < 0.0001). Among them, the difference in the maximum dose to the spinal cord between VMAT_PRO_ and VMAT_PO_ was remarkable (9.04 Gy vs. 11.08 Gy with *p* < 0.0001). For Ring_1.5 cm_, no significant difference in V_115%_ for VMAT_PRO_ and VMAT_PO_ was observed. The values of V_105%_ and V_110%_ for VMAT_PRO_ were much smaller than those for VMAT_PO_, with the differences being statistically significant (0.44 cm^3^vs. 2.66 cm^3^ with *p* < 0.001 for V_105%_, and 0.02 cm^3^vs. 0.63 cm^3^ with *p* = 0.039 for V_110%_).

Table 3 summarizes the average DV parameters of both VMAT_PRO_ and VMAT_PO_ for the lumbar spine cases. For the PTVs, all of the DV parameters showed significant differences between VMAT_PRO_ and VMAT_PO_, except for the minimum dose (*p* = 0.207). Similar to the DV parameters of the PTVs for cervical and thoracic spine cases, the values of D_5%_, D_2%_, maximum dose, and mean dose were lower in VMAT_PRO_ than in VMAT_PO_. In contrast, the values of D_98%_ and D_90%_ were slightly higher for VMAT_PRO_ than for VMAT_PO_, demonstrating that VMAT_PRO_ exhibited coverage and uniformity of the dose to the PTV. In the same vein, the target conformity and dose homogeneity in the PTVs of VMAT_PRO_ were better than those of VMAT_PO_, with statistical significance (0.92 vs. 0.86 with *p* < 0.0001 for CI_paddick_ and 0.26 vs. 0.29 with *p* < 0.0001 for HI).

**Table 3:** Average dose-volumetric parameters of the planning target volume, cauda equina, planning organ at risk volume of cauda equina, and 1.5-cm ring structure surrounding PTV for lumbar spine stereotactic ablative radiotherapy plans

DV parameter	PRO	PO	*p*-value
PTV			
Volume (cm^3^)	83.52 ± 46.66		-
D_98%_ (Gy)	14.49 ± 1.54	14.30 ± 1.50	0.015
D_90%_ (Gy)	17.45 ± 0.32	17.20 ± 0.45	< 0.0001
D_5%_ (Gy)	19.07 ± 0.24	19.49 ± 0.48	< 0.0001
D_2%_ (Gy)	19.21 ± 0.26	19.66 ± 0.51	< 0.0001
Maximum dose (Gy)	20.12 ± 0.43	20.55 ± 0.77	< 0.001
Minimum dose (Gy)	8.68 ± 2.04	8.45 ± 2.30	0.207
Mean dose (Gy)	18.22 ± 0.06	18.41 ± 0.17	< 0.0001
CI	0.92 ± 0.07	0.86 ± 0.10	< 0.0001
HI	0.26 ± 0.10	0.29 ± 0.10	< 0.0001
Cauda equina			
Volume (cm^3^)	6.24 ± 2.08		-
D_1.5 cc_ (Gy)	6.04 ± 1.42	6.94 ± 1.75	< 0.0001
D_0.5 cc_ (Gy)	8.30 ± 1.51	9.39 ± 1.54	< 0.0001
D_0.1 cc_ (Gy)	10.37 ± 1.51	11.58 ± 1.47	< 0.0001
D_0.035 cc_ (Gy)	11.19 ± 1.45	12.40 ± 1.38	< 0.0001
Maximum dose (Gy)	12.93 ± 1.36	14.10 ± 1.21	< 0.0001
Mean dose (Gy)	4.56 ± 0.79	5.13 ± 1.01	< 0.0001
Cauda equina PRV			
Volume (cm^3^)	10.08 ± 3.44		-
D_1.5 cc_ (Gy)	8.39 ± 1.57	9.23 ± 1.68	< 0.0001
D_0.5 cc_ (Gy)	10.74 ± 1.47	11.71 ± 1.43	< 0.0001
D_0.1 cc_ (Gy)	12.64 ± 1.33	13.53 ± 1.25	< 0.0001
D_0.035 cc_ (Gy)	13.33 ± 1.25	14.17 ± 1.15	< 0.0001
Maximum dose (Gy)	14.82 ± 1.17	15.52 ± 1.07	< 0.0001
Mean dose (Gy)	5.10 ± 0.85	5.60 ± 1.01	< 0.0001
Ring_1.5 cm_			
V_105%_ (cm^3^)	1.03 ± 1.30	3.20 ± 3.29	< 0.0001
V_110%_ (cm^3^)	0.09 ± 0.21	0.46 ± 0.79	0.008
V_115%_ (cm^3^)	0.00 ± 0.01	0.02 ± 0.07	0.155

Note: DV = dose-volumetric; PRO = progressive resolution optimizer algorithm; PO = photon optimizer algorithm; PTV = planning target volume; D_n%_ = dose received by at least *n*% volume of the planning target volume; CI = conformity index; HI = homogeneity index; D_n cc_ = dose received by at least *n* cc volume of the planning target volume; PRV = planning organ at risk volume; V_n%_ = absolute volume of a structure irradiated by at least *n*% of the prescription dose; Ring_1.5 cm_ = 1.5-cm ring structure surrounding PTV

For the cauda equina and the corresponding PRVs, all of the DV parameters for VMAT_PRO_ were considerably smaller than those for VMAT_PO_, showing statistically significant differences (all *p* < 0.0001). In particular, the difference in D_0.035 cc_ of the cauda equina between VMAT_PRO_ and VMAT_PO_ was remarkable (11.19 Gy vs. 12.40 Gy with *p* < 0.0001). For Ring_1.5 cm_, V_115%_ showed no significant differences between VMAT_PRO_ and VMAT_PO_. Similar to the DV parameters of the PTVs for the cervical and thoracic spine cases, the values of V_105%_, and V_110%_ for VMAT_PRO_ were much smaller than those for VMAT_PO_, with the differences being statistically significant (1.03 cm^3^vs. 3.20 cm^3^ with *p* < 0.0001 for V_105%_, and 0.09 cm^3^vs. 0.46 cm^3^ with *p* = 0.008 for V_110%_).

**Fig. 1:**
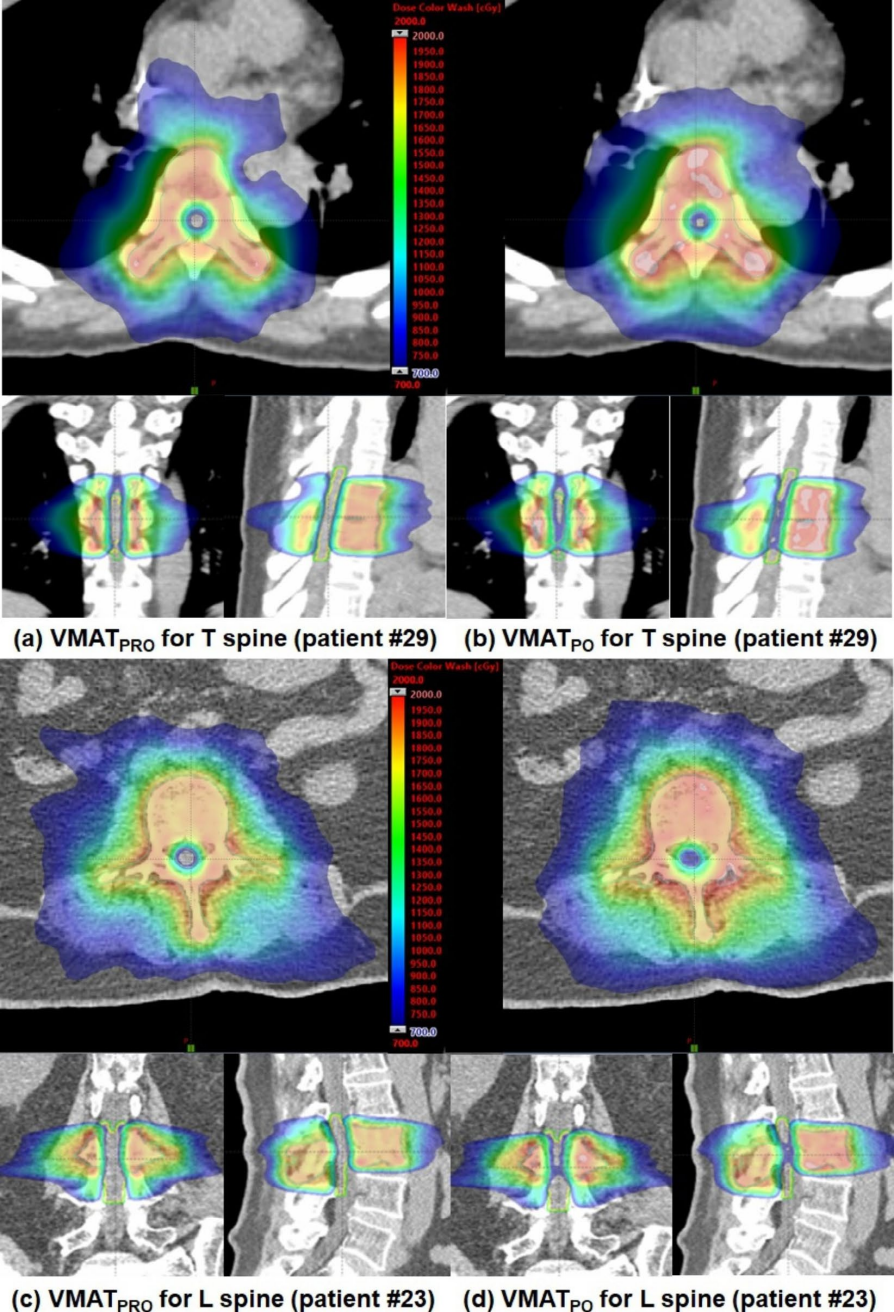
Representative dose distributions of spine stereotactic ablative radiotherapy cases (patient #29 and #23) for thoracic (T) and lumbar (L) spines, respectively, are shown. Dose distributions of volumetric modulated arc therapy plans generated by a progressive resolution optimizer (VMAR_PRO_) (a) and by a photon optimizer (VMAT_PO_) (b) for patient #29 are shown. For patient #23, dose distributions of VMAT_PRO_ (c) and VMAT_PO_ (d) are also shown. Doses are depicted by color wash with 7 Gy (the lowest dose) in blue, and 20 Gy (maximum dose) in red. The lowest dose, 7 Gy, was chosen to represent the minimum doses inducing the radiation myelopathy

**Fig. 2:**
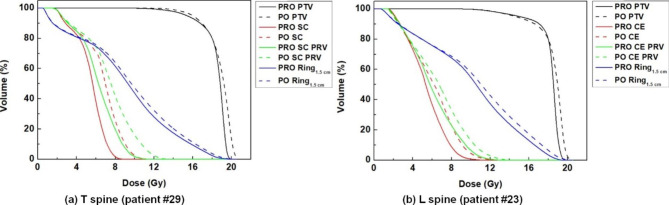
Representative dose-volume histograms of spine stereotactic ablative radiotherapy (SABR) cases (patient #29 and #23) for thoracic (T) spine (a) and lumbar (L) spine (b), respectively, are shown. A progressive resolution optimizer (PRO), and photon optimizer (PO) are plotted with the solid and dashed lines, respectively, for planning target volume (PTV), spinal cord (SC), the corresponding planning organ-at-risk volume (SC PRV), cauda equine (CE), the corresponding PRV (CE PRV), and 1.5-cm ring structure surrounding PTV (Ring_1.5 cm_)

Overall, the use of the PRO algorithm for generating VMAT plans could provide better target coverage and sparing of normal tissues surrounding the target volumes for spine SABR. For the dosimetric evaluation, the dose distributions of VMAT_PRO_ and VMAT_PO_ from a representative patient are shown in Fig. 1. The dose-volume histograms for this patient are shown in Fig. 2.

### Total MU and MCS_v_

The average total MUs and MCS_v_ values are listed in Table 4. In the cervical and thoracic spine SABR cases, the PRO algorithm generated more complex VMAT plans with significantly higher total MUs than the PO algorithm, showing statistical significance (6020.4 vs. 4850.1 with *p* < 0.0001 for total MUs and 0.389 vs. 0.495 with *p* < 0.0001 for MCS_v_). Similarly, the lumbar spine SABR showed higher total MUs and modulation for VMAT_PRO_ than VMAT_PO_ (6267.8 vs. 5038.2 with *p* < 0.0001 for total MUs and 0.425 vs. 0.528 with *p* < 0.0001 for MCS_v_).

**Table 4:** Average total monitor units and modulation complexity score for volumetric modulated arc therapy for cervical and thoracic, and lumbar spine stereotactic ablative radiotherapy plans

	PRO	PO	*p*-value
	Cervical and thoracic spine		
MU	6020.4 ± 608.6	4850.1 ± 594.52	< 0.0001
MCS_v_	0.389 ± 0.082	0.495 ± 0.119	< 0.0001
	Lumbar spine		
MU	6267.8 ± 469.3	5038.2 ± 386.8	< 0.0001
MCS_v_	0.425 ± 0.053	0.528 ± 0.085	< 0.0001

Note: PRO, progressive resolution optimizer algorithm; PO, photon optimizer algorithm; MU, monitor unit; MCS_v_ modulation complexity score for volumetric modulated arc therapy proposed by Masi et al. al. (2013)

### Correlation of DV parameters with total MU and MCSv

The values of the correlation coefficient (*r*) and corresponding *p*-values of Δ in the DV parameters with total MUs and MCS_v_ for cervical and thoracic spine SABR are shown in Table 5. In general, Δ in the DV parameters of the spinal cord and spinal cord PRV showed a strong correlation with Δ in the total MUs and MCS_v_, except for the Ring_1.5 cm_ (with p < 0.05). The values of *r* of ΔD_1.2 cc_ and Δmean dose of spinal cord, and ΔD_0.035 cc_ and Δmean dose of spinal cord PRV for ΔMU and ΔMCS_v_ were larger than 0.69, and 0.79, respectively, with statistical significance (*p* < 0.001).

**Table 5:** Correlation coefficients (*r*) with corresponding *p*-values of Pearson’s or Spearman’s correlation test of dose-volumetric parameters of organ-at-risk volumes to modulation complexity score for volumetric modulated radiation therapy and total monitor units for cervical and thoracic spine stereotactic ablative radiotherapy plans

	ΔMU		ΔMCS_v_	
DV parameter	*r*	*p*	*r*	*p*
Spinal cord				
ΔD_1.2 cc_	-0.679	< 0.0001	0.793	< 0.0001
ΔD_0.35 cc_	-0.504	0.007	0.644	< 0.001
ΔD_0.035 cc_	-0.349	0.069	0.380	0.047
ΔMaximum dose	-0.394	0.039	0.584	0.001
ΔMean dose	-0.686	< 0.001	0.790	< 0.0001
Spinal cord PRV				
ΔD_1.2 cc_	-0.533	0.004	0.605	0.001
ΔD_0.35 cc_	-0.287	0.138	0.460	0.015
ΔD_0.035 cc_	-0.652	< 0.001	0.781	< 0.0001
ΔMaximum dose	-0.276	0.155	0.540	0.003
ΔMean dose	-0.635	< 0.001	0.804	< 0.0001
Ring_1.5 cm_				
ΔV_105%_ (cm^3^)	-0.075	0.704	0.216	0.269
ΔV_110%_ (cm^3^)	-0.004	0.983	0.133	0.499
ΔV_115%_ (cm^3^)	-0.089	0.652	0.137	0.487

Note: Δ = differences in the values (PO minus PRO) between the two algorithms, MU = monitor unit, MCS_v_ = modulation complexity score for volumetric modulated arc therapy proposed by Masi et al. (2013). DV = dose-volumetric, D_n cc_ = dose received by at least *n* cc volume of the planning target volume, PRV = planning organ at risk volume, V_n%_ = absolute volume of a structure irradiated by at least *n*% of the prescription dose, Ring_1.5 cm_ = 1.5-cm ring structure surrounding PTV

The values of *r* and the corresponding *p*-values of Δ in the DV parameters with total MUs and MCS_v_ for lumbar spine SABR are shown in Table 6. Overall, Δ in the DV parameters of the spinal cord and spinal cord PRV showed a moderate correlation with Δ in the total MUs and MCS_v_, except for the Δmaximum dose of the cauda equina, ΔD_0.1 cc_, ΔD_0.035 cc_, and Δmaximum dose of the cauda equina PRV ( p < 0.05). For the Δmean dose of the cauda equina, the maximum values of *r* with *p* values less than 0.0001 were observed (-0.802 for ΔMU and 0.834 for ΔMCS_v_).

**Table 6:** Correlation coefficients (*r*) with corresponding *p*-values of Pearson’s and Spearman’s correlation test of dose-volumetric parameters of organ-at-risk volumes to modulation complexity score for volumetric modulated radiation therapy and total monitor units for lumbar stereotactic ablative radiotherapy plans

	ΔMU		ΔMCS_v_	
DV parameter	*r*	*p*	*r*	*p*
Cauda equine				
ΔD_1.5 cc_	-0.675	< 0.0001	0.674	< 0.0001
ΔD_0.5 cc_	-0.549	0.002	0.569	0.002
ΔD_0.1 cc_	-0.535	0.003	0.515	0.005
ΔD_0.035 cc_	-0.537	0.003	0.514	0.005
ΔMaximum dose	-0.298	0.116	0.260	0.173
ΔMean dose	-0.802	< 0.0001	0.834	< 0.0001
Cauda equine PRV				
ΔD_1.5 cc_	-0.505	0.006	0.529	0.004
ΔD_0.5 cc_	-0.406	0.030	0.439	0.018
ΔD_0.1 cc_	-0.307	0.105	0.323	0.088
ΔD_0.035 cc_	-0.255	0.181	0.245	0.200
ΔMaximum dose	0.035	0.857	-0.025	0.899
ΔMean dose	-0.736	< 0.0001	0.774	< 0.0001
Ring_1.5 cm_				
ΔV_105%_ (cm^3^)	-0.717	< 0.0001	0.601	0.001
ΔV_110%_ (cm^3^)	-0.697	< 0.0001	0.562	0.002
ΔV_115%_ (cm^3^)	-0.457	0.013	0.350	0.063

Note: Δ = differences in the values (PO minus PRO) between the two algorithms, MU = monitor unit, MCS_v_ = modulation complexity score for volumetric modulated arc therapy proposed by Masi et al. (2013). DV = dose-volumetric, D_n cc_ = dose received by at least *n* cc volume of the planning target volume, PRV = planning organ at risk volume, V_n%_ = absolute volume of a structure irradiated by at least *n*% of the prescription dose, Ring_1.5 cm_ = 1.5-cm ring structure surrounding PTV

## Discussion

In this study, we evaluated the performance of the PRO and PO algorithms for generating spine SABR VMAT plans by comparing the DV parameters of the target volume and surrounding normal tissues, total MU, and MCS_v_. To date, this study is the first attempt to assess the plan quality of VMAT_PRO_ and VMAT_PO_ in patients with cervical, thoracic, and lumbar spinal tumors. When comparing the DV parameters of the target volume and surrounding normal tissues, VMAT_PRO_ achieved better PTV coverage and dose uniformity while reducing the dose to the spinal cord or cauda equina and Ring_1.5 cm_ than VMAT_PO_. However, for VMAT_PRO_, improvements in plan dosimetric quality can lead to increases in overall plan complexity and total MUs, which can compromise treatment deliverability and efficiency, respectively.

Similar studies have investigated the optimization algorithms in terms of dosimetric quality for various SABR treatment sites, such as the lungs and brain [17–19]. Visak et al. investigated the dosimetric quality of VMAT_PRO_ and VMAT_PO_ in 12 lung SABR patients with a single dose of 30 Gy [18]. They demonstrated that the PRO algorithm provided higher MUs and higher modulation of lung SABR VMAT plans, while the dose to normal tissues was reduced compared with the PO algorithm. They also reported that the PO algorithm increased the intermediate-dose spillage, which can result from more exposure to normal tissues [18]. In contrast, some institutions demonstrated that the plan quality between both algorithms was comparable with no statistical significance, although VMAT_PRO_ increased the total MUs and plan complexity [17, 19]. For the prostate, head and neck, and brain treatment sites, which do not involve SABR VMAT plans, there was an increase in the total MUs and the level of modulation when the PO algorithm was used, showing better OAR sparing and an opposite result to the SABR VMAT plans [16, 20]. Thus, the efficacy of the optimization algorithms may vary depending on the radiotherapy regimen or treatment site. Therefore, it is necessary to analyze the plan quality for each condition.

**Fig. 3:**
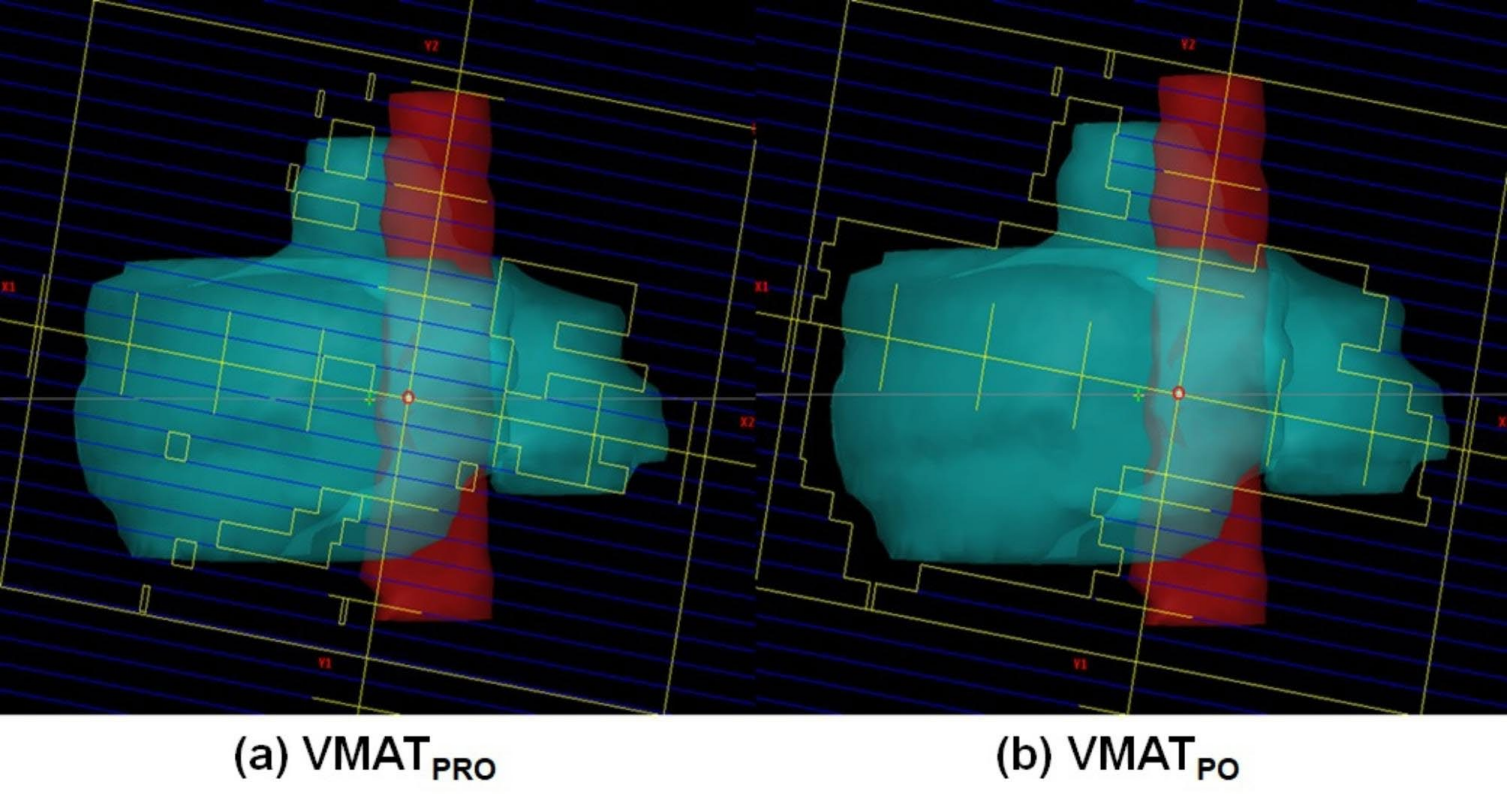
Multi-leaf collimator openings for randomly selected control points between volumetric modulated arc therapy plans generated by a progressive resolution optimizer (VMAT_PRO_) (a) and by a photon optimizer (VMAT_PO_) (b) for a representative spine stereotactic ablative radiotherapy patient

The PRO algorithm, which utilizes a point-cloud model, can have a high number of calculation points (1) inside small or narrow structures, such as lenses, optic nerves, and spinal cords, or (2) around the edge of irregular structures, such as vertebral bodies and head and neck nodes [25]. In addition, the grid size for the structure can be adjusted as much as the user wants, resulting in an increase in the calculation points inside the structure. Because this facilitates more degrees of freedom for the calculation grid size, a sophisticated modulation scheme of VMAT plans is possible using the PRO algorithm [16]. With these characteristics, the PRO algorithm can generate many small and irregular MLC openings compared with the PO algorithm. In contrast, the PO algorithm uses only one fixed grid size with a single matrix over the CT images during optimization, and the degrees of freedom for the calculation grid size are relatively small compared with the PRO algorithm. The matrix resolution of PO algorithm can be selected from three options (Fine, Normal, and Fast) of 1.25, 2.5, and 5 mm, respectively. This study used the Normal resolution rather than the Fine resolution because it required huge amount of computer memory and long computation time. There were no studies evaluating the effect of the matrix resolution of PO algorithm on the optimization performance. Empirically, changes in the matrix resolution below 2.5 mm did not show the dosimetric differences when a few spine cases were tested for our study. The PO algorithm tends to significantly remove small openings when compared with the PRO algorithm [17]. The MLC openings for randomly selected control points between VMAT_PRO_ and VMAT_PO_ for a representative spine SABR patient are shown in Fig. 3. It was observed that MLC shapes defined by the PRO algorithm were smaller and more irregular than those defined by the PO algorithm, and were associated with sparing critical normal organs during optimization. Spine SABR has the characteristic of having a long and narrow OAR including the spinal cord or cauda equina that must be protected within an irregular PTV of the vertebral body. Limiting the number of calculation points per volume leads to a potential loss of information that must be considered during optimization. To effectively reduce the dose to the spinal cord or cauda equina, which are widely recognized as critical organs, during optimization, the use of the PRO algorithm would be more advantageous for spine SABR VMAT plans because of the ability to generate small or irregular MLC shapes, and the greater degrees of freedom for the calculation grid size. For the dosimetric evaluation, the dose distributions in VMAT_PRO_ and VMAT_PO_ for spine SABR are shown in Fig. 1. A noticeable dose reduction in the spinal cord or cauda equina for VMAT_PRO_ was achieved compared with VMAT_PO_ that had good PTV coverage.

Until now, the latest released version of Varian Eclipse is 16.2. Varian has announced that PRO algorithm is no longer supported from version 16.0 onwards and has no improved functions up to the version 16.0. On the other hand, PO algorithm has been continuously improving its features until 16.2. Among them, the most representative feature to affect the optimization performance is the aperture size controller (ASC) released in version 15.5. With this function, the users are allowed to control the field aperture shape, which results in increasing the field size and then decreasing the complexity of the MLC apertures. The users can adjust the complexity of the plan generated PO algorithm through the ASC function, which may lead to different results from the version of the plan used in our study. However, even before ASC was developed, it was demonstrated that PO algorithm (ver. 13.7) had a tendency to generate simpler field apertures than PRO algorithm shown in Fig. 3. For this reason, it is likely to show similar results for the dosimetric comparison between PRO and PO algorithms, regardless of version of algorithm. Nevertheless, This will be investigated in future studies because no planning study of VMAT for spine SABR generated using different versions of PO algorithms has been performed.

To improve the dosimetric plan quality, high modulation, implying complex MLC movements and the usage of small or irregular MLC apertures, is required; however, this leads to an increase in the number of total MUs and a decrease in plan delivery accuracy [16, 26]. Liu et al. compared PRO and PO algorithms in terms of plan quality and correlations between gamma passing rates and plan complexities for both lung SABR and brain SRS [17]. The criteria for the gamma analysis were 3%/3 mm and 2%/2 mm for lung SABR and 5%/1 mm and 3%/1 mm for brain SRS, with 10% as the threshold value. They reported less agreement between planned and delivered dose distributions when VMAT_PRO_ had higher MLC variability and total MUs. Although the overall gamma passing rates with all gamma criteria for VMAT_PRO_ decreased compared with those for VMAT_PO_, the average gamma passing rates for lung SABR and brain SRS were above 90% and 95% under the criteria of 2%/2 mm and 3%/1 mm and 3%/3 mm and 5%/1 mm, respectively, and VMAT_PRO_ was considered clinically acceptable [17]. In this regard, our institution acquired the gamma passing rates of portal dosimetry with gamma criteria of 2%/1 mm, and all VMAT_PRO_ and VMAT_PO_ (> 90%) were found to be clinically acceptable. Additionally, our previous study investigated the correlation between gamma passing rates and the modulation degree of VMAT plans [27]. We utilized identical TrueBeam STx with a high-definition 120™ MLC and then generated 100 VMAT plans with various tumor sites, including the lung, spine, liver, brain, and head and neck, using the PRO algorithm. Measurements of the dose distributions for each VMAT plan were acquired using MapCHECK2™ and ArcCHECK™ (Sun Nuclear Corporation, Melbourne, FL, USA). As a result, the average gamma passing rates for all criteria were above 90%, which is regarded as clinically acceptable. It was found that there was less correlation between gamma passing rates with all criteria and MCS_v_, with no statistical significance, except for the correlation of the 3%/3 mm criterion with ArcCHECK™ (*r* = 0.210, *p*-value = 0.036) [27]. Thus, we can conclude that TrueBeam STx has a guaranteed performance with a high degree of agreement between the planned and actual delivered doses, regardless of plan complexity. For the TrueBeam STx used in this study, the modulation degree of the VMAT plans according to the optimization algorithm could be considered less important. Nevertheless, careful evaluation of its deliverability as well as that of other treatment machines is needed for the clinical implementation of the PRO algorithm.

Radiation myelopathy is a rare but catastrophic complication of radiation exposure to the spinal cord or cauda equine [1–3]. The RTOG 0631 guidelines recommend dose constraints to the spinal cord (D_1.2 cc_ < 7 Gy, D_0.35 cc_ < 10 Gy, and D_0.035 cc_ < 14 Gy) and cauda equina (D_5 cc_ < 14 Gy, and D_0.035 cc_ < 16 Gy) for spine SABR [28]. However, according to the retrospective study by Sahgal et al., the maximum dose of the spinal cord in a single fraction was estimated, ranging from 9.20 to 12.40 Gy which was associated with a 1–5% risk of radiation myelopathy [12, 29]. For the cervical and thoracic spine SABR in our study, the maximum dose to the spinal cord for VMAT_PRO_ (9.04 Gy) was approximately 2 Gy less than that for VMAT_PO_ (11.08 Gy), resulting in a 2% reduction in the risk of radiation myelopathy. The maximum dose of spinal cord PRV for VMAT_PO_ (13.83 Gy) exceeded 12.40 Gy while that for VMAT_PRO_ (12.25 Gy) did not. Since radiation myelopathy can lead to death, it is important to reduce the risk of this complication by sparing the spinal cord or cauda equina as much as possible. Therefore, patients treated with spine SABR may benefit from VMAT_PRO_.

## Conclusions

The use of VMAT_PRO_ resulted in improved coverage and uniformity of dose to the PTV, as well as OARs sparing, compared with that of VMAT_PO_ for cervical, thoracic, and lumbar spine SABR. Better dosimetric plan quality generated by the PRO algorithm was observed to result in higher total MUs and plan complexity. Therefore, careful evaluation of its deliverability should be performed with caution during the routine use of the PRO algorithm.

## Electronic supplementary material

Below is the link to the electronic supplementary material.

Supplementary Material 1

## Data Availability

The datasets used and/or analysed during the current study are available from the corresponding author on reasonable request and have been provided to the journal as supplementary material.

## List of Abbreviations

SABR: Stereotactic ablative radiotherapy
VMAT: Volumetric modulated arc therapy
MLC: Multi-leaf collimator
OAR: Organ at risk
IMRT: Intensity modulated radiotherapy
DVH: Dose-volume histogram
TPS: Treatment planning system
PO: Photon optimizer
DVO: Dose-volume optimizer
PRO: Progressvie resolution optimizer
MU: Monitor unit
SRS: Stereotatic radiosurgery
VMAT_PRO_: PRO-generated VMAT plan
VMAT_PO_: PO-generated VMAT plan
CT: Computed tomography
PTV: Planning target volume
CTV: Clinical target volume
PRV: Planning organ-at-risk volume
Ring_1.5 cm_: 1.5-cm ring structure surrounding the PTV
RTOG: Radiation Therapy Oncology Group
NTO: Normal tissue optimization
MCS_v_: Modulation complexity score for the VMAT
*r*: Correlation coefficient
